# Antibacterial PEGylated Solid Lipid Microparticles for Cosmeceutical Purpose: Formulation, Characterization, and Efficacy Evaluation

**DOI:** 10.3390/ma13092073

**Published:** 2020-04-30

**Authors:** Giuseppe Angellotti, Denise Murgia, Alessandro Presentato, Maria Cristina D’Oca, Amalia Giulia Scarpaci, Rosa Alduina, Maria Valeria Raimondi, Viviana De Caro

**Affiliations:** 1 Dipartimento di Discipline Chirurgiche, Oncologiche e Stomatologiche, Università degli Studi di Palermo, 90127 Palermo, Italy; giuseppe.angellotti@unipa.it (G.A.); denise.murgia@unipa.it (D.M.); 2 Dipartimento di Scienze e Tecnologie Biologiche Chimiche e Farmaceutiche (STEBICEF) Università degli Studi di Palermo, 90128 Palermo, Italy; alessandro.presentato@unipa.it (A.P.); amaliagiuliascarpaci@gmail.com (A.G.S.); valeria.alduina@unipa.it (R.A.); mariavaleria.raimondi@unipa.it (M.V.R.); 3 Dipartimento di Fisica e Chimica, Università degli Studi Palermo, 90128 Palermo, Italy; mariacristina.doca@unipa.it

**Keywords:** solid lipid microparticles, lipid microspheres, Resveratrol, Limonene, 1-Hexadecanol, Labrasol, antimicrobial activity, tape-stripping, *Staphylococcus aureus* ATCC 25923, porcine ears

## Abstract

The development of efficacious means of delivering antioxidant polyphenols from natural sources for the treatment of skin diseases is of great interest for many cosmetic and pharmaceutical companies. Resveratrol (RSV) and Limonene (LIM) have been shown to possess good anti-inflammatory and antibacterial properties against *Staphylococcus aureus* infections responsible for many skin disorders, such as acne vulgaris. In this study, solid lipid microparticles are designed as composite vehicles capable of encapsulating a high amount of trans-RSV and enhancing its absorption through the stratum corneum. A microparticulate system based on mixture of PEGylate lipids, long-chain alcohols and LIM is able to entrap RSV in an amorphous state, increasing its half-life and avoiding inactivation due to isomerization phenomena, which represents the main drawback in topical formulations. Particles have been characterized in term of shape, size distribution and drug loading. Antimicrobial tests against *S. aureus* have highlighted that empty microspheres possess per se antimicrobial activity, which is enhanced by the presence of LIM, demonstrating that they can represent an interesting bactericide vehicle for RSV administration on the skin.

## 1. Introduction

Skincare is one of the major concerns of modern society, since having healthy skin is synonymous with beauty, health, and youth. Furthermore, the look of the facial skin influences interpersonal relationships, because those who have skin with imperfections and blemishes are seen as unappealing, compared to those who have smooth skin [1]. Some of these imperfections are due to bacterial infections; for instance, acne vulgaris is the most common of all skin diseases. It includes the formation of comedones, papules, pustules, nodules, and cysts, following the obstruction and inflammation of the pilosebaceous units (hair follicles and their corresponding sebaceous glands). The onset of acne lesions relies on (i) the altered follicular keratinization, leading to comedones; (ii) the increased and altered sebum production; (iii) the follicular colonization by *Propionibacterium acnes*; and (iv) complex inflammatory mechanisms, which involve both innate and acquired immunity [2]. Moreover, cellulitis, erysipelas, impetigo, folliculitis, furuncles, and carbuncles are also common skin diseases caused by *Staphylococcus aureus* infection [3]. Currently, these infections are treated with both oral and topical antibacterial agents. Even though they are widely used, the prolonged use of chemotherapeutics is the main cause of the spread of antibacterial resistance phenomena in microorganisms, leading to infectious-disease-treatment failure worldwide [4,5]. As a consequence, an additional strategy to contrast antibiotic resistance is the research of new molecules targeting biofilm formation [6,7]. During the last few years, the attention toward the Resveratrol (RSV) has been increasing considerably, due to its proven beneficial properties on human health [8]. RSV (3,4′, 5-trihydroxy-trans-stilbene) is a polyphenol phytoalexin obtained from red grapes, red wine and wild berries, as well as through both chemical and biotechnological synthesis (performed by engineered bacterial and yeast strains) [9]. RSV exists in two isomeric forms (3,4′, 5-trihydroxy-*cis*-stilbene and 3,4′, 5-trihydroxy-*trans*-stilbene), but only the *trans* one possesses antioxidant and anti-inflammatory activity. The inactive *cis* isoform derives from isomerization of trans RSV, due to the UV radiation, alkaline pH and high temperature [10]. RSV has been shown to be active against a wide spectrum of bacterial strains (i.e., *Neisseria gonorrhoeae*, *Neisseria meningitidis*, *Helicobacter pylori*, *Staphylococcus aureus*, *Enterococcus faecalis*, *Pseudomonas aeruginosa*, *Proteus mirabilis*, and *Propionibacterium acnes*) [11,12]. It has been shown that RSV can inhibit the expression of IL-8, ICAM-1, VCAM-1, and neutrophil adhesion by TNF-α stimulation, as well as the release of TNF-α by mast cells through histamine. Moreover, RSV reduces the pro-inflammatory signal, avoiding adenosine release from activated platelets and inhibiting protease-activated (PAR) and P2 receptors by interfering with mitogen-activated protein (MAPK) and c-Jun (JNK) kinases [13].

R-(+)-limonene (LIM)—or 1,8-p-menthadiene-1-methyl-4-(1-methylethenyl)-cyclohexene—is a terpenoid compound contained in essential oils of citrus peel [14], which has gained considerable interest for its remarkable biological activity, since it holds antioxidant [15], antitumor [16], anti-inflammatory [17] and antimicrobial activity [18]. It is worth mentioning that LIM is also widely used as a flavor in commercial products, such as perfumes, detergent and soap. Thus, the development of innovative formulations that take advantage of the biologically relevant properties of RSV and LIM compounds appears to be of paramount importance. On this matter, these compounds can be used in association with common antibacterial agents, to reduce the limitations related to their prolonged use, increasing, at the same time, their effectiveness through elicited synergistic effects [19]. However, conventional cosmetic products containing RSV are not able to guarantee a massive absorption into the skin, whose main drawback is ascribed to the low aqueous solubility of RSV itself, therefore precluding the possibility of loading a fairly sufficient amount of RSV in such products to benefit of its properties. On the same line, even LIM is difficult to incorporate in a conventional pharmacological formulation, since it can be lost due to the high vapor pressure typical of terpenoid substances. In this context, lipid microparticles have emerged as attractive vectors for pharmaceutical and cosmetic topical applications due to their advantages compared to polymer systems, representing also an innovative approach to overcome the abovementioned drawbacks related to the instability of bioactive compounds. Specifically, the encapsulation of the latter in lipid microparticles based on pegylated glycerides and/or long-chain fatty alcohols can enhance the stability and bioavailability of the compounds themselves [20].

Here, lipid microspheres loaded with RSV and LIM—homogeneously dispersed in a molecular form—were developed and characterized. This system was designed to guarantee an optimal active absorption once applied on the skin, enhancing the antioxidant, anti-inflammatory, and antibacterial properties of both RSV and LIM. The innovative characteristics of the product may lead to its possible application as a cosmeceutical product for skincare.

## 2. Materials and Methods

### 2.1. Materials

Trans-Resveratrol (RSV), α-Cyclodextrin (α-CD), β-Cyclodextrin (β-CD), Glyceryl Stearate (35-50 Stearate), and Soy Lecithin were purchased from A.C.E.F. (Fiorenzuola D’Arda, Italy); Labrasol (PEG-8 Caprylic/Capric Glycerides), Gelucire 62/05, Labrafil M-1944, Labrafil M-2130, Isostearyl Isostearate, and Tefose 1500 were kindly supplied from Gattefossè (Saint-Priest, France); 1-Hexadecanol (EXA), R-(+)-Limonene (LIM), and Bovine Serum Albumin (BSA) were purchased from Sigma-Aldrich Chemie (Steinheim, Germany). Dimethyl sulfoxide (DMSO) was purchased from VWR International (Milan, Italy). Phosphate Buffer Saline pH 7.4 (PBS) was prepared by dissolving 2.80 g of KH_2_PO_4_ and 20.5 g of Na_2_HPO_4_ in 1 L of distilled water. Then, 0.9% saline solution was prepared by dissolving 9 g of sodium chloride (NaCl) in 1 L of distilled water. The citrate buffer solution pH 5.5 was prepared by dissolving 10.26 g of anhydrous sodium citrate and 6.35 g of citric acid monohydrate in 500 mL of distilled water.

All chemicals and solvents were analytical grade and purchased from VWR International (Milan, Italy) and used without further purification.

Porcine ears were kindly supplied by the Municipal Slaughterhouse of Villabate (Palermo, Italy). The experimental data were processed using Kaleidagraph v.3.5 (Synergy Software Inc., Reading, Citybatches were prepared by varying the lipids ratio and the RSV content. Microspheres containing RSV and LIM were named MC-RL. Microspheres either containing only RSV (MC-R) or LIM (MC-L), as well as those empty (MC-E) were prepared by the same method. Six batches of each formulation were prepared for the studies.

### 2.2. Determination of Microspheres’ Melting Temperature Range

The melting temperature range of all microspheres batches was investigated by using a Buchi melting point apparatus model B-540. Briefly, the sample was placed in a capillary glass and analyzed at a heating rate of 5 °C/min, until microspheres entirely melted.

### 2.3. Microspheres Characterization

#### 2.3.1. Morphology Analysis

Microspheres were observed with an optical-scanning microscope (Leica DFC 420C, Wetzlar, Germany) connected to a digital camera (Photometrics, Tucson, AZ, USA), evaluating their morphological characteristics, quality, shape, size, and homogeneity. The images were analyzed by Image-Pro Plus.

#### 2.3.2. Microspheres’ Size Distribution Analysis

The separation of the microspheres into various size fractions was carried out, using an Endecotts Octagon 200 test sieve shaker (Endecotts Ltd., London, UK) and standard mesh wire sieves (Endecotts Ltd., London, UK). Microspheres were placed on top of a stack of six standard stainless-steel sieves, having mesh sizes between 90 and 300 μm, and assembled in descending order. The sieves were mechanically shaken in continuous for 20 min. Then, microparticles were carefully collected and weighted to determine their size distribution, which was calculated according to the following equation:

(1) $$R\%=\frac{Pf}{Pt}\times 100$$

where *Pf* and *Pt* are the weight of microsphere remained on a determined sieve and the total weight of microspheres analyzed, respectively. The results were expressed as mean ± SD of six batches. The fractions of microspheres having a size between 100 and 300 μm were collected and considered suitable for further studies.

#### 2.3.3. RSV Encapsulation Efficacy (EE) and Drug Loading (DL) Assay

The RSV incorporated into the microspheres was measured by spectrophotometric quantitative determination (UV-Vis Shimadzu PharmaSpec 1700 model, Shimadzu, Tokyo, Japan). Randomly selected microspheres aliquots (5 mg) were transferred into 100 mL flasks, completed to volume by methanol, and sonicated (Branson B 1200 cleaner, Branson Ultrasonic Corporation, Danbury, CT, USA), allowing the release of the incorporated RSV.

The amount of RSV loaded into the microspheres was measured at λ_max_ = 305 nm and based on a calibration curve, which was constructed by analyzing standard RSV solutions ranging from 0.001 to 0.005 mg/mL (regression equation Abs = 0.04 + 143.4x (mg/mL); R^2^ = 0.998).

The encapsulation efficacy (EE) and drug loading (DL) were calculated according to the following equations:

(2) $$EE\%=\frac{Wenc}{Wtot}\times 100$$

(3) $$DL\%=\frac{Wenc}{Wlipid}\times 100$$

where *Wenc* is the amount of RSV quantified into the microspheres, whereas *Wtot* and *Wlipid* are the amount of RSV and the amount of lipid mixture initially mixed, respectively. The analysis was performed in triplicate.

#### 2.3.4. Limonene Detection by Gas Chromatography–Flame Ionization Detector (GC–FID) Analysis

GC–FID analyses were performed on a Shimadzu GC 2010A equipped with a SUPELCOWAX^®^ 10 column (30 m × 0.25 mm I.D., 0.25 μm film thickness, Supelco, Bellefonte, PA, USA) and a FID detector, according to the following thermic program: from 60 °C (held for 3 min) to 130 °C with 15 °C/min ramp rate, then up to 240 °C (held for 1 min). Helium (He, 99.9% pure), as a carrier gas, was used at a velocity of 25.9 cm/s. Injector and FID temperatures were set at 250 and 280 °C, respectively.

#### 2.3.5. Differential Scanning Calorimetry (DSC) Analysis

DSC analysis was performed, using a Setaram DSC131 EVO (Caluire, France). Samples (about 8 mg) were inserted in aluminum crucibles and heated up to 300 °C, at a heating rate of 10 °C/min and continuous nitrogen flow of 1 mL/min.

#### 2.3.6. Electron Paramagnetic Resonance (EPR) Spectroscopy Analysis

To evaluate if paramagnetic center of RSV was not modified during the MC-RL preparation, the form and parameters of EPR spectra of RSV and MC-RL were compared. EPR measurements were carried out on 50 mg of RSV, MC-RL, and MC-E at room temperature, using a Bruker ELEXSYS E-500 spectrometer (Billerica, MA, USA) operated at 9.8 GHz (X-band), operating at microwave power 38 mW and modulation amplitude 0.7 mT.

#### 2.3.7. RSV Detection by High-Performance Liquid Chromatography (HPLC) Analysis

HPLC analyses were performed with a Shimadzu LC-10AD VP instrument (Tokyo, Japan) equipped with a binary pump LC-10AD VP, a UV SPD-M20A diode array detector (DAD), a 20 µL injector, and a computer integrating apparatus (EZ Start 7.4 software, Shimadzu Scientific Instruments, Inc., Columbia, MD, USA). Chromatographic separation was achieved on a reversed-phase column ACE^®^ EXCEL 5 C18-AMIDE (5 µm, 4.6 mm × 125 mm, Aberdeen, UK), using a mobile phase consisting of trifluoroacetic acid in water (0.01% *v/v*) (A) and methanol (B). For separation, the gradient method was developed according to the following mobile phase (A:B) ratios: 99.5:0.05→0–3 min, 10:90→3–7 min, 10:90→7–13 min, 99.5:0.05→13–18 min.

The flow rate was set at 1 mL/min, the UV wavelength range was 200–700 nm, and set at 306 nm to identification of RSV. In these conditions, the retention time of RSV was 10.6 min.

### 2.4. Photostability Evaluation of RSV Entrapped into the Microspheres

Loaded MC-RLs (100 mg) and free RSV powder were aliquoted in glass vials and exposed to direct natural light for 70 days. During that time (days), 5 mg aliquots were solubilized in methanol, to evaluate RSV content by both UV-Vis spectrophotometer and HPLC analyses. Experiment was performed in triplicate.

### 2.5. Ex-Vivo Permeation Assay throughout Porcine Ear Skin

Porcine ears were sampled from 8-month-old domestic pigs post-sacrifice, being stored in PBS (pH 7.4) until further processing. Within 1 h of sampling, ear specimens were transferred to the laboratory in a refrigerated transport box, cleaned, and excised. The fat was removed from the dermal surface, and the tissue from each ear was divided into 2 pieces of approximately 6 cm^2^. Some specimens were freshly used, while others were submerged in a 5% trehalose solution as a cryoprotectant for 30 min, being then blotted dry with soft tissue and stored at −80 °C, for up to 6 months. The specimen was thawed in PBS solution before the experiments, since the stratum corneum layer is not influenced by storage conditions [21].

#### 2.5.1. Preliminary RSV Solubility Tests

To enhance the aqueous solubility of the RSV, maintaining the sink conditions of the system, preliminarily various acceptor fluids were evaluated. PBS buffer solution supplemented with α-CD 0.5% *w/v*, or β-CD 2% *w/v*, or BSA 5% *w/v*, or DMSO 10% *v/v*, and citrate buffer solution pH 5.5 amended with β-CD 2% *w/v* were prepared. Increasing amounts of RSV were added to 1 mL of each solution, until saturation was reached. The suspension was kept in a thermostatic water bath (Heidolph MR3001K Hotplate Stirrer with Heidolph EXT3001 Temperature Probe, Heidolph Instruments, Schwabach, Germany) at 32 °C and stirred for 120 min. The samples were then centrifuged for 15 min at 15.000 rpm (mySPIN™ 12 Mini Centrifuge, Thermo Fisher Scientific, Waltham, MA, USA), to remove any precipitate. The supernatant was collected, diluted in methanol, and quantified by a UV-Vis spectrophotometer. The experiments were performed in triplicate.

#### 2.5.2. Evaluation of the Tissue Permeation by Vertical Franz Cell Apparatus

The permeation of RSV throughout the epidermis of porcine ear skin was evaluated, using Franz-type diffusion cell (Permeagear, flat flange joint, 9 mm orifice diameter, 16 mL acceptor volume, SES GmbH-Analysesysteme, Bechenheim, Germany), used as a two-compartment open model [22]. The ears’ epidermis was obtained by dipping it for approximately 1 min in a warm (60 °C) saline solution; therefore, the epidermis was heat-detached from the underlying tissues [23], obtaining 2.16 ± 0.20 mm thickness epithelia, which was measured using a digital micrometer (Vogel, Germany). The heat treatment does not compromise the epithelia integrity and permeability [23]. Before starting the test, the tissue was equilibrated in an isotonic solution for 12 h, to remove any enzymes that could interfere with drug analyses. To do so, the acceptor compartment was filled with PBS and 2% β-CD, whereas the donor compartment with PBS only. Epithelium was placed between the 2 compartments, holding the system for 15 min at 35 ± 0.5 °C. Then, PBS of the donor compartment was substituted with either a 1 mL solution of propylene glycol-buffer citrate pH 5.5 (in a ratio of 4:6) containing 1 mg of RSV or 10 mg of MC-RL microspheres. The acceptor fluid was stirred at 100 rpm to maintain the homogeneity of solution. Every 30 min, 0.5 mL of acceptor fluid was withdrawn and replaced with fresh fluid, to maintain the sink condition. RSV was quantified by UV-Vis analyses based on a calibration curve in PBS with 2% β-CD (Validation parameters: λ_max_ = 305 nm, range 0.0005–0.0075 mg/mL, regression equation Abs = −0.024 + 123.72x (mg/mL) and R^2^ = 0.999).

At each sampling point (30, 60, and 90 min), the Franz cell was disassembled, and the porcine epithelium was analyzed to evaluate the amount of RSV entrapped into the tissue [24]. Specifically, each epithelium was washed 3 times with distilled water (2 mL), being then dipped in 1.5 mL of methanol heated up to 50 °C. The extraction was repeated three times and the collected liquors were transferred in a 5 mL flask, and brought to volume. The amount of RSV extracted was quantified as stated above. The experiments were performed in triplicate for each time point.

#### 2.5.3. Evaluation of Tissue Penetration by Tape-Stripping Assay

The tape-stripping assay was used to analyze the RSV drug penetration into the stratum corneum of porcine epithelial model [25,26]. Briefly, the test involves the removal of the stratum corneum cell layer through adhesive tape. The porcine ear epidermis was cropped to obtain rectangular parts (about 4 cm × 3 cm) and equilibrated with PBS for 15 min at 35 °C. A stencil was placed over the tissue, which leaves a rectangular 2 cm^2^ surface of skin uncovered, to standardize the application surface. Then, 15 mg of MC-RLs was applied gently by rubbing with a saturated glove finger until melting (about 1 min). After an application time of 30 min, any unabsorbed formulation residue was removed, and the tape-stripping procedure was started (Scotch Book tape No. 854 3M, 3M Italia Srl, Milan, Italy)), using a roller, assuring tape strips’ adhesion with a homogeneous pressure to the skin. A total of 10 applications to the skin were performed (named as S1, S2, S3, S4, S5, S6, S7, S8, S9, and S10). The amount of RSV entrapped was extracted in methanol (3 × 3 mL), at 60 °C, and quantified after transferring the liquor collected a 10 mL flask, which was brought to volume with the same solvent. The amount of drug extracted was determined by UV-Vis analysis. Data were reported as mean of six experiments.

### 2.6. Antibacterial Activity Evaluation of Microspheres

MC-RLs, MC-Rs, MC-Ls, and empty microspheres (MC-E) were tested for their antimicrobial efficacy against the indicator pathogen strain *Staphylococcus aureus* ATCC 25923, by using a disc diffusion antibiotic sensitivity assays as described in the literature [27,28]. A concentrated *S. aureus* cell suspension (about 10^9^ Colony Forming Unit per milliliter of culture (CFU/mL)) was spread on Luria Bertani (hereafter named as LB and composed of (g/L): NaCl (10), tryptone (10), yeast extract (5), and bacteriological agar (15)) agar plates. Afterward, five different dilutions (0.90, 2.25, 4.05, 5.85, and 7.88 mg/mL) of each microsphere sample were prepared, starting from stock solutions (18 mg/mL) in dimethyl sulfoxide (DMSO). From these dilutions, 20 μL—corresponding to 18, 45, 81, 117, or 158 μg of microspheres—were deposited on LB agar plates and air-dried, being then the plates incubated for 24 h at 37 °C. Bacterial growth inhibition was evidenced by the formation of a clear zone, where the microspheres had been deposited. DMSO (20 μL) was used as a negative control. Experiments were performed in triplicate.

### 2.7. Data Analysis

All data are presented as means (±SE, Standard Error) of at least three replicates (n = 3). Significant differences among means from replicate analyses (*p* < 0.05) were determined by Student’s *t*-test or One-Way ANOVA followed by Dunnett’s multiple comparison tests. The level of significance was set at *p* < 0.05 for all statistical tests.

## 3. Results and Discussion

Resveratrol is a natural stilbene compound that holds relevant pharmacological and cosmetic properties, such as antioxidant, anti-keratogenesis, and anti-angiogenic activity, being particularly suitable in counteracting bacterial skin infections due to its anti-inflammatory and antimicrobial activity [29,30].

When it comes to the development of RSV-based pharmaceutical or cosmetic formulations, it must be considered that the physicochemical properties of RSV (i.e., low solubility and chemical instability) largely impair its inclusion in conventional semi-solid topical products. Nevertheless, the RSV partition coefficient (LogP = 3.09) [31] and good solubility in lipid excipients allow its use to generate formulations that rely on solid lipid microparticles (SLM). The latter are also ideal to encapsulate extremely volatile substances, such as LIM, considering that essential oils extracted from *Citrus* species containing a high percentage of LIM (85%) were shown to have antimicrobial effects against acne-inducing bacteria [32], as well as to promote the absorption of other substances through the human skin and mucous membrane [33,34]. Thus, SLMs containing RSV and LIM are here proposed as an innovative formulation capable of exerting a synergistic antimicrobial efficacy, which might promote the absorption of various drugs.

### 3.1. Screening of Lipids Excipient

Lipophilic excipients must have peculiar features from a physicochemical perspective (i.e., solid at room temperature, chemically inert, high capacity to incorporate drugs, and capable of protecting the latter from physicochemical degradation phenomena) [35] and a biological point of view (i.e., biocompatible, neither carcinogenic or teratogens nor allergenic, and poorly oxidizable) [36] to use them for generating both pharmaceutical and cosmetic formulations. Here, 1-Hexadecanol (EXA) as non-toxic, biocompatible, biodegradable long-chain fatty alcohol was chosen as a solid lipid, as its surfactant characteristic allows this excipient to behave as an emulsifier agent, avoiding the need for further surfactant addition usually required for SLMs preparation using melt dispersion method [13,37]. In addition, EXA was shown a certain effectiveness in the inhibition of *S. aureus* growth [38]. The liquid lipid Labrasol was chosen due to its ability to solubilize up to 10% *w/w* of RSV, while other lipids failed in solubilizing even 5% *w/w* of RSV, as experimentally evaluated and highlighted in Table 1.

Finally, LIM was used as an enhancer for RSV absorption, as well as to give a pleasant odor to the formulation.

### 3.2. Preparation of RSV and LIM Solid Lipid Microspheres

MC-RLs were prepared according to the melt dispersion technique [34,35], as this method is highly reproducible, avoiding the use of organic solvents and, therefore, the risk of finding toxic residues in the formulation. Briefly, the technique involves-cooling induced solidification of the oily phase of a two-phase system. The drug and the lipid mixture were mixed in a homogeneous melt and emulsified into the aqueous external phase; solid spherical, free-flowing microspheres were formed after rapid cooling [36].

During the MC-RL preparation, the stirring speed was a key factor to obtaining microspheres with a suitable size for the drug topical administration. Optimal speed was detected in 700 rpm, since higher speed produced very small microspheres that could not be recovered once filtered; instead, lower speed caused the formation of large (ø > 300 μm) and non-spherical particles. Several MC-RL formulations were synthesized by modifying EXA, Labrasol, LIM, and RSV ratios, whose melting temperature range is reported in Table 2, below.

MC-RLs’ melting temperature range was influenced by different EXA and Labrasol ratios and RSV content, while LIM did not seem to play such an effect. The yield of microspheres for each batch was about 77.5%. The expected RSV transcutaneous absorption mechanism occurred by microspheres’ melting, and since the microspheres’ softening temperature was above that of the skin (35 °C), once applied to the skin, a slight finger pressure to melt them completely was needed. On the other hand, a too-low melting temperature leads to obtain microspheres difficult to be stored under environmental conditions. Moreover, MC-RL3 showed to quickly melt once rubbed on the skin. Thus, further characterizations were performed on MC-RL3.

### 3.3. Microspheres Characterization

#### 3.3.1. Morphology Analysis and Particle Size Distribution

**Figure 1:**
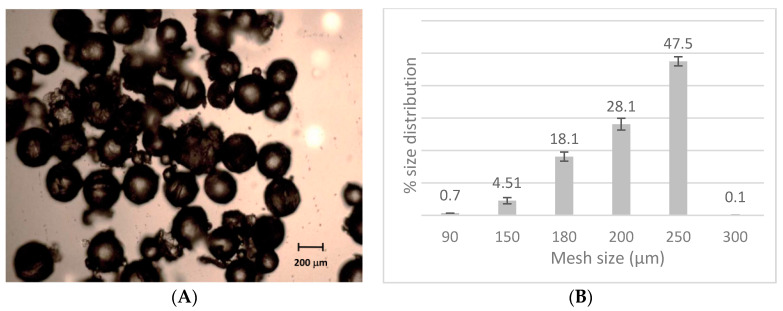
Microspheres’ morphology (**A**) and percent size distribution (**B**). Data are presented as mean ± SE (n = 6).

Microscopy observations unveiled that microspheres were spherical and regular shaped (Figure 1A), confirming the preparation method’s effectiveness. Moreover, this morphological aspect is of paramount importance, as round-shaped microparticles possess a high sliding capacity and manageability. Granulometric analysis showed that the most represented microsphere population had a size distribution centered at 250 μm (47.5%), while smaller microparticles were present in a lower amount as the dimension decreased (Figure 1B). Thus, the sphericity, as well as the mean obtained for the selected formulation for the ongoing permeation studies, was considered as appropriate for the topical administration, as microparticles should be handled like powder and melt by finger pressure.

#### 3.3.2. Drugs Loading Determination

The encapsulation efficacy (EE) and drug loading (DL) of RSV were found to be 84.7% and 6.35%, respectively. In MC-RL, the solid lipid matrix encloses oil section in which drug solubility is considerably high increasing total drug loading capacity. Therefore, the liquid pegylated-lipid present in the microspheres largely affects RSV entrapment efficiency by creating imperfections in a highly ordered crystal matrix, consequently providing sufficient space for a large amount of drug to lodge. The high EE is attributed to RSV high partition coefficient and its poor water solubility, which guarantees low drug loss during the preparation, confirming the good reproducibility and reliability of the method.

As regards the LIM loading, it was not possible to quantify it. In fact, given the small amount initially used, the GC–FID analyses allowed to detect its presence, which however was below the limit of quantitation. Nevertheless, its presence inside the microspheres was confirmed by the strong smell that emanated when they were applied to the skin.

#### 3.3.3. DSC, HPLC, and EPR Analyses Evaluation

**Figure 2:**
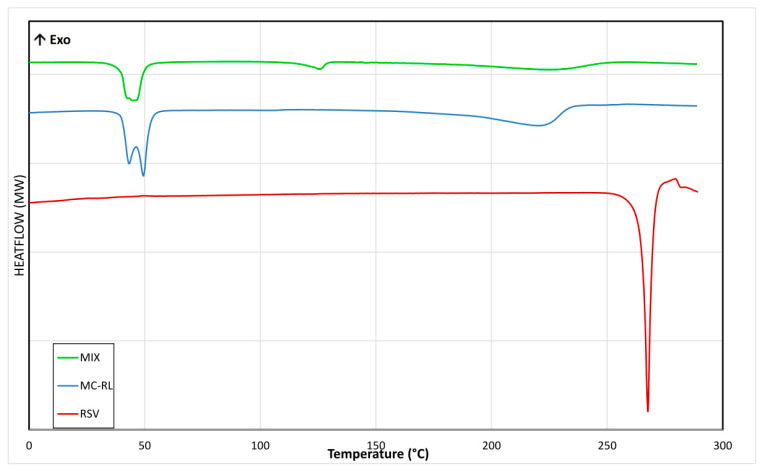
DSC thermograms of RSV (red line), MC-RL (blue line), and MIX (green line).

Crystalline or amorphous state of RSV inside microspheres was investigated by DSC analysis. Thermograms of pure RSV, MC-RL, and the mixture containing the lipids and RSV (named MIX) in the same ratios to prepare MC-RL, previously melted (to mix them) and then cooled, are reported on Figure 2. The RSV thermogram highlighted a well-defined endothermic peak at 267 °C, ascribed to its melting point, which was absent in both MIX and MC-RL thermograms.

MC-RL and MIX thermograms showed endothermic peaks at 43 and 49 °C ascribed to lipid components, being absent that of RSV, which underlined its total amorphization. Furthermore, the MC-RL thermogram presented a broad peak between 200 and 240 °C. Thus, to evaluate whether either RSV decomposition or its cis isomerization occurred, EPR and HPLC–DAD spectroscopy analyses were performed.

The EPR is a technique used to detect and study chemical species with unpaired electrons, and then with a paramagnetic center, such as antioxidant compounds. This method gives information about the interactions with other nuclei around unpaired electrons, providing information on the structure, by calculation of parameters of the EPR spectra: amplitude peak–peak (H_pp_), linewidths (ΔB_pp_), and g-factors. Amplitude H_pp_ increases with the increasing of paramagnetic center concentration in the sample. ΔB_pp_ and g-factors depend on molecular structure of the samples and magnetic interactions in the chemical units.

By comparing the shape and parameters of EPR spectra of pure RSV and MC-RL, the displacement of the paramagnetic center of RSV, an indicator of its possible degradation during the preparation process of MC-RL, was assessed. Moreover, the EPR spectrum of MC-E was acquired to verify that the EPR signal of MC-RL was not due to microsphere excipients.

**Figure 3:**
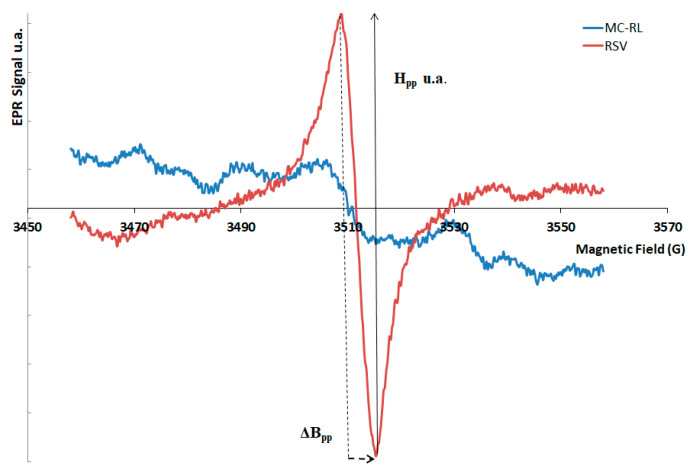
EPR spectra of RSV and MC-RL. The H_pp_ and ΔB_pp_ parameters are shown.

The EPR spectra of RSV (Figure 3) showed a single peak; the amplitude H_pp_ of singlet was calculated by the sum in absolute value of the maximum and minimum of the peak, corresponding to 0.455 u.a. ΔB_pp_ is the width of the RSV single peak and corresponds to 7 G.

The g-factor of peak was calculated using the following equation:

(4) $$g=\frac{hv}{\mu_{B}B}$$

where h = Planck constant, ν = microware frequency, μ_B_ = Bohr Magnetone, and B = Magnetic Field.

Moreover, the EPR spectrum of MC-RL is a singlet (Figure 3); the ΔB_pp_ and g-factor were calculated of single peak of MC-RL and correspond to same value of RSV: ΔB_pp_ = 7 G and g-value = 2.0037. These results permit us to ascertain that the paramagnetic center was the same and it was not modified during the MC-RL preparation. The H_pp_ value of singlet of MC-RL was calculated and corresponds to 0.056 u.a. The low value of H_pp_ results from the RSV amount in the microspheres (6.35%), rather than from a lower concentration of paramagnetic centers. On the EPR spectra of MC-E, no EPR signal is detected (not significantly different from background noise), indicating that the excipients of microspheres do not have paramagnetic centers.

The EPR analysis results highlighting how the paramagnetic center of RSV was not modified during the microspheres preparation demonstrate the RSV antioxidant capacity not been impaired.

**Figure 4:**
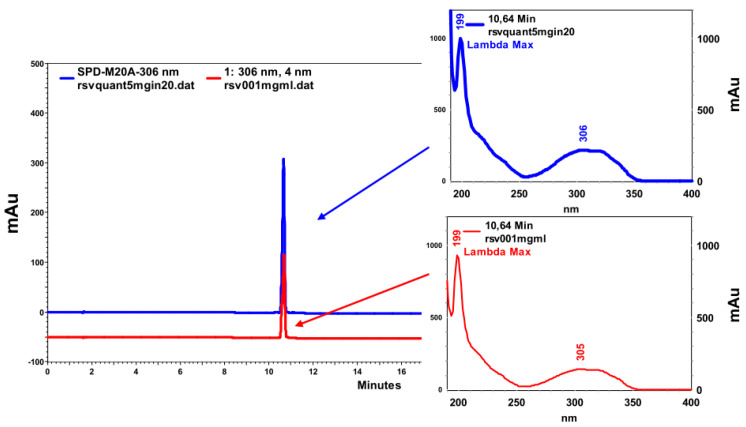
Comparison between the chromatograms of the RSV standard (red) and the microspheres (blue), with the respective UV-Vis absorption spectra.

For HPLC-DAD analysis, a standard solution (0.01 mg/mL) of RSV and a MC-RL solution containing comparable amount of RSV were eluted, and the results were compared. MC-RL and RSV chromatograms revealed the same sharp peak at Rt of 10.6 min, having identical UV-Vis absorption spectra with the maximum absorption peak at 306 nm (Figure 4) and the absence of absorption peaks from 400 to 700 nm. Moreover, the microsphere sample showed no presence of degradation products at wavelengths between 200 and 700 nm [39]. This evidence ruled out any doubt on the maintenance of RSV stability during the production process.

### 3.4. Stability Evaluation of RSV Microencapsulate to the Light Exposure

**Figure 5:**
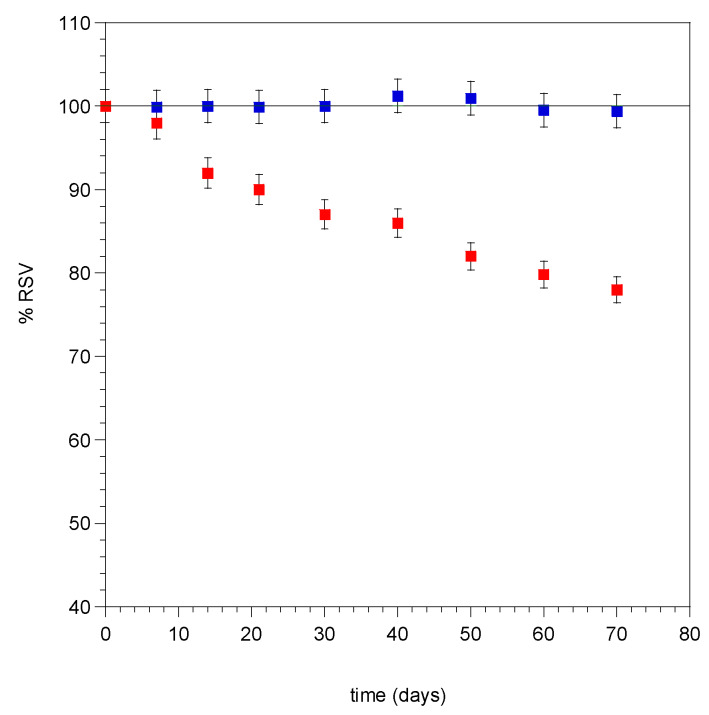
Percentage of the decay of trans-RSV, free (■) and microencapsulated (■) after light exposure. Values are presented as mean ± SE (n = 3).

It is well-known that trans-RSV is highly photosensitive and rapidly suffers isomerization to cis-form, followed by degradation, when exposed to ultraviolet or visible light [39]. Photosensitivity and poor solubility are the main drawbacks impairing the use of RSV in cosmetic and pharmaceutical products. Microencapsulation should overcome these drawbacks and allows an enhancement of RSV absorption in the tissue. For this reason, the protective effect of the encapsulation of RSV against photodegradation in comparison to RSV-free powder was evaluated. As a result, microencapsulated RSV exposed to natural light was protected from photodegradation phenomena over a period of two months, as highlighted in Figure 5.

### 3.5. Ex-Vivo Permeation Assays

Porcine ear skin represents the best animal model suitable to simulate human skin for ex vivo permeation studies [40]. Indeed, this epithelial tissue shares physiological characteristics with human one such as a subcutaneous layer tissue, the permeable epidermis layer, follicular structure, and the number of ear gland-piliferous [41,42,43].

#### 3.5.1. Evaluation of RSV Solubility in Aqueous Solution

According to the definition of a cosmetic product, no drug or functional substance within the formulation must reach the systemic circulation; hence, the ability of RSV present in both solution and microspheres to cross the heat-separated porcine epidermis was tested by the Franz type cell assay [44]. Since the RSV has very low solubility in water of 0.05 mg/mL [45], several buffer solutions containing solubilizing agents were tested, to increase the RSV concentration gradient on the sides of the epidermis, as it represents the main driving force for the passive diffusion of drugs (Table 3).

Based on the obtained results (Table 3), PBS amended with 2% β-CD was chosen as the acceptor liquid for the permeation test, to maximize the concentration gradient.

#### 3.5.2. Franz Cell Assay

The ability of free RSV to accumulate in the epidermis was analyzed by performing ex vivo permeation studies, using vertical Franz type diffusion and comparing the behavior of the designed formulation in promoting the RSV diffusion into the epithelium, as well as favoring its penetration rather than the permeation up to the systemic circulation. Due to its poor aqueous solubility, to ensure that 1 mg of RSV was completely solubilized, the donor compartment was filled with RSV (1 mg/mL) in propylene glycol:buffer citrate pH 5.5 in a ratio of 4:6. The use of propylene glycol has been found necessary to maintain solubilized RSV during the experiments in an amount equivalent to those contained in MC-RLs [45]. In the case of both RSV and MC-RLs, the UV-Vis analyses of collected fluids from the acceptor camber showed that the RSV was not detectable in this compartment, suggesting that RSV was not capable of crossing the epithelium, according to cosmetic product definition.

**Figure 6:**
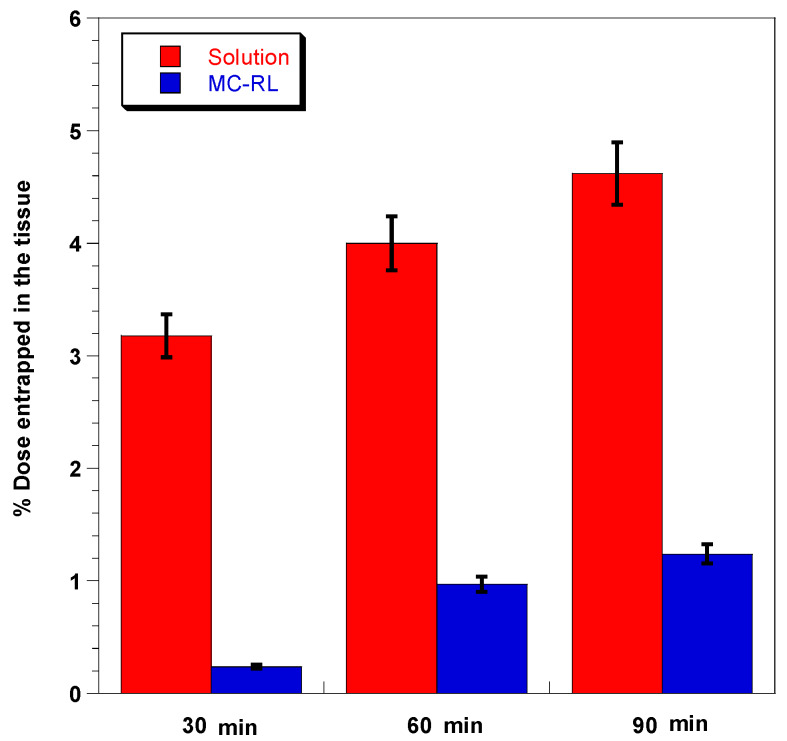
Comparison between RSV extracted from the epithelium, after application of solution (red) and MC-RL (blue) in the different application times. Data are presented as mean ± SE (n = 3).

The evaluation of RSV amount accumulated in the epithelium was carried out at the end of the permeation assay, underlining how RSV can accumulate in the epithelium increasing during time; however, this accumulation trend is slower when it is carried out by the microspheres (Figure 6), likely due to the experimental conditions adopted. Indeed, microspheres have a softening temperature between 39 and 44 °C; therefore, the lack of a manual application could impair MC-RLs’ melting and, successively, RSV release and permeation into the epithelium. On the other hand, free RSV can permeate the epithelium thanks to the vehicle (propylene glycol), which acts as an absorption enhancer.

Moreover, the low percentage values of RSV accumulated in the membrane are attributable to the histology of the external porcine auricular tissue, which has a structure conformation and thickness that offers a high resistance to the RSV absorption [43].

#### 3.5.3. Tape-Stripping Assay

**Figure 7:**
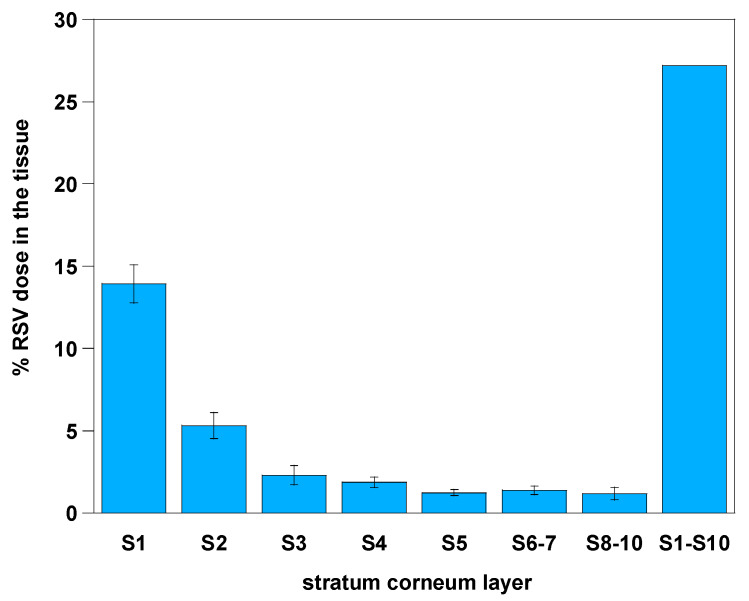
Percentage of RSV dose entrapped in skin layers. Data are presented as mean ± SE (n = 6).

The microspheres were also analyzed by the tape-stripping method [26], to either confirm or refuse the results obtained through Franz cell assay. The results in Figure 7 are shown as the mean of six different tests carried out with painstaking care.

As a result, the dose of RSV in each tape strip, which represents the amount of RSV present in each stratum corneum layer, highlights that the accumulation into the membrane is 20% greater than the data collected by the Franz cell assay; in particular, the average amount of RSV accumulated is 27.2% of the applied dose, of which 21.5% is accumulated in the three most external layers.

### 3.6. Antibacterial Activity against Staphylococcus Aureus ATCC 25923

**Figure 8:**
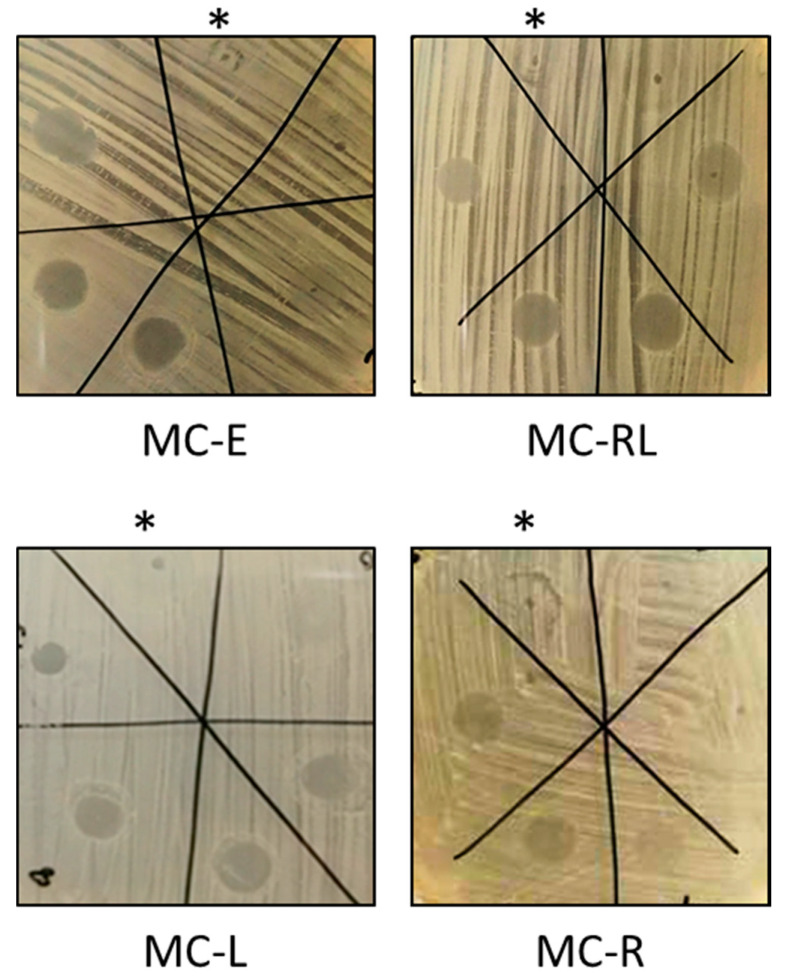
Microbiological assays showing antibacterial activity of 0, 18, 45, 81, 117, or 158 μg of microspheres. MC-E, MC-RL, MC-R, and MC-L indicate empty MC or loaded MC with RSV/LIM, RSV or LIM. The samples were deposited clockwise, and the asterisk indicates the sample containing 20 μL of DMSO and no MCs. Where the MC was deposited, a regular and circular inhibition halo was visible if cells did not grow.

Since LIM and RSV are known for inhibiting bacterial growth [6,7,8,9,10,11,12,13], the MC-E, MC-RL, MC-R, and MC-L were analyzed for their antibacterial activity against *S. aureus* by microbiological assays (Figure 8). These assays demonstrated that 81 μg of MC-E display antibacterial activity against *S. aureus* (Figure 8). The loading of LIM into MC made the MC-L more potent than MC-E, since 45 μg was enough to inhibit bacterial growth; differently, the addition of RSV to MC did not have the same extent, since the antibacterial activity of MC-R was as high as MC-E (81 μg). This is probably due to the low amount of RSV loaded into MC that is lower than the already reported Minimal Inhibitory Concentration (MIC). Indeed, the loaded RSV into MC-R should be around 4.86 μg (in 20 µL, equal to 243 μg/mL). Although no concordant results of the MIC (100 [27] or 1000 µg/mL [28]) of RSV against *S. aureus* have been reported so far, the MC-loaded RSV amounts are lower than the possible lowest MIC (100 µg/mL). The reason for controversial data of MIC of RSV could be dependent on the experimental conditions, specifically the growth medium. Here, LB-agar was used as growth medium for which the MIC of RSV against *S. aureus* was 1000 µg/mL [28]. As expected, MC-RL was as active as MC-L against *S. aureus*, since RSV did not synergistically contribute to the antimicrobial activity. Notwithstanding, the loading of RSV into the MC-RL might be useful, since better antibacterial properties were reported for RSV in respect to benzoyl peroxide, which is conventionally used to treat acne [6], and the procedures of loading could be optimized to increase RSV amount.

## 4. Conclusions

In this work, a nanocomposite system consisting of solid lipid microparticles containing Resveratrol and R-(+)-limonene, dispersed internally at the nanometric level in its amorphous state, was developed and studied to unveil its ability to counteract common skin infections caused by pathogen bacterial strains (i.e., *Staphylococcus aureus* and *Propionibacterium acnes*). Microparticles have proven their suitability as a drug-delivery vehicle for skin administration of RSV, allowing its high encapsulation efficiency within SLM in an amorphous state, as well as promoting its absorption through the stratum corneum of porcine epidermis. Furthermore, SLM increases the half-life of RSV itself by protecting it from physicochemical factors and avoiding isomerization phenomena, which would inexorably lead to a pharmacological inactive form of RSV, when incorporated into conventional cosmetic creams. Furthermore, the formulated lipid microspheres possess per se antimicrobial activity, which is enhanced by the presence of LIM demonstrating that they can represent an interesting bactericidal vehicle for drug administration on the skin. Although RSV does not appear to enhance the SLM antimicrobial activity, it can display its beneficial effects (i.e., antioxidant, antiaging, and anti-inflammatory) once penetrated in the stratum corneum.

## Figures and Tables

**Table 1:** RSV dissolution in different liquid lipophilic excipients.

Excipient	RSV (5% *w/w*)	RSV (10% *w/w*)	RSV (15% *w/w*)
Gelucire 62/05	Opalescent	Opalescent	Opalescent
Glyceryl Monostearate	Opalescent	Opalescent	Opalescent
Isostearyl Isostearate	Opalescent	Opalescent	Opalescent
Labrafil M-1944	Opalescent	Opalescent	Opalescent
Labrafil M-2130	Opalescent	Opalescent	Opalescent
Labrasol	Clear	Clear	Slightly Opalescent
Soy Lecithin	Opalescent	Opalescent	Opalescent
Tefose 1500	Clear	Slightly Opalescent	Opalescent

**Table 2:** Composition of mixtures used for microspheres preparation with relative melting temperature range (m.t.r.) and MTR of microspheres obtained.

Sample	EXA (g)	Labrasol (g)	RSV (g)	LIM (μL)	Mixture M.t.r. (°C)	Microspheres M.t.r. (°C)
MC-RL1	0.57	0.38	0.05	10	45–51	44–47
MC-RL2	0.555	0.37	0.075	10	47–53	43–49
MC-RL3	0.462	0.462	0.075	10	43–49	39–44
MC-R3	0.462	0.462	0.075	−	43–49	39–44
MC-L3	0.500	0.500	−	10	38–42	36–40
MC-E	0.500	0.500	−	−	38–42	36–40

**Table 3:** RSV solubility in buffer solutions. The results are presented as mean ± SE (n = 3).

Aqueous Solvents	Solubility (mg/mL)
PBS + α-CD 0.5%	0.153 ± 0.008
PBS + BSA 5%	0.442 ± 0.035
PBS + β-CD 2%	0.886 ± 0.011
PBS + DMSO 10%	0.194 ± 0.010
Citrate buffer (pH 6.2) + β-CD 2%	0.803 ± 0.003
